# Establishment of a rapeseed meal fermentation model for iturin A production by *Bacillus amyloliquefaciens* CX‐20

**DOI:** 10.1111/1751-7915.13483

**Published:** 2019-09-30

**Authors:** Wenchao Chen, Xuli Ma, Xiuzhen Wang, Shouwen Chen, Anna Rogiewicz, Bogdan Slominski, Xia Wan, Fenghong Huang

**Affiliations:** ^1^ Oil Crops Research Institute of the Chinese Academy of Agricultural Sciences Wuhan 430062 China; ^2^ Key Laboratory of Biology and Genetic Improvement of Oil Crops Ministry of Agriculture Wuhan 430062 China; ^3^ Oil Crops and Lipids Process Technology National & Local Joint Engineering Laboratory Wuhan 430062 China; ^4^ Hubei Key Laboratory of Lipid Chemistry and Nutrition Wuhan 430062 China; ^5^ Hubei Collaborative Innovation Center for Green Transformation of Bio‐Resources Environmental Microbial Technology Center of Hubei Province College of Life Sciences Hubei University Wuhan 430062 China; ^6^ Department of Animal Science Faculty of Agricultural and Food Sciences University of Manitoba Winnipeg MB R3T 2N2 Canada

## Abstract

Iturin A is an important broad‐spectrum antifungal cyclic lipopeptide used as an ideal potential biological control agent. However, its application is limited mainly due to the producer strains’ low productivity and the high production costs. Here, a potentially industrial strain *Bacillus amyloliquefaciens* CX‐20 was proved to use low‐cost rapeseed meal (RSM) as the sole source of all nutrients except the carbon source for the high productivity of iturin A. A fermentation model was first established to analyse the specific roles of different RSM components on iturin A production. Proteins and minerals in RSM were confirmed to play positive role, whereas fibre had negative effect. And the maximal concentration of iturin A was predicted to be more than 1.64 g l^−1^ by the established evaluation model. Moreover, submerged fermentation of *B. amyloliquefaciens* CX‐20 demonstrated a strong ability to hydrolyse RSM and release water‐soluble nutrients. This fermentation broth, a mixture of *Bacillus*, iturin A and RSM hydrolysate, could simultaneously combat clubroot disease and promote the growth of *Brassica napus*. In conclusion, this study provides a promising strategy to achieve full utilization of RSM for the production of a combination of value‐added biological control agent and biofertilizer.

## Introduction

Rapeseed meal (RSM), which is a by‐product of oil extraction from rapeseed, consists mainly of proteins, carbohydrates and minerals (Wang *et al*., 2010; Lomascolo *et al*., 2012). As the second most widely produced and inexpensive by‐product after soya bean meal (SBM), effective usage of RSM can play a crucial role in the sustainable bio‐economy. It has been the objective of various studies over recent decades, especially as the current price of SBM is continuing to rise. The application of RSM hydrolysate as a feedstock for microbial bioconversions, produced using filamentous fungi in solid‐state fermentation (SSF), has been intensively investigated (García *et al*., 2013; Kiran *et al*., 2013; Chatzifragkou *et al*., 2014). However, RSM pretreatment with fungi in SSF has the drawbacks of low homogeneity of mass and heat transfer, inconvenient handling, difficult monitoring, additional time and cost, environmental pollution by a strong odour and the residue waste (Mizumoto *et al*., 2007; Chen *et al*., 2011). Therefore, it is of great significance for the valorization of RSM to screen microorganisms that can directly utilize the RSM nutrients in submerged fermentation to produce high‐value‐added metabolites.

Different *Bacillus* spp., especially *B. subtilis* and *B. amyloliquefaciens*, have been used as cell factories for the production of many enzymes for the hydrolysis of proteins, carbohydrates and lipids in raw materials such as RSM (Ramos *et al*., 2011; Liang *et al*., 2015). Moreover, direct utilization of untreated RSM as a nitrogen source for iturin A production by *B. subtilis* 3–10 in submerged fermentation was firstly proved feasible by our team (Jin *et al*., 2014), although the iturin A production was low. Iturin A is arguably the most well studied broad‐spectrum antifungal cyclic lipopeptide. It consists of a cyclic heptapeptide linked to a 14 to 17‐carbon β‐amino fatty acid chain (Ongena and Jacques, 2008). Its special molecular structure that enables it to change the permeability and integrity of the cell membrane makes it the principal antifungal substance responsible for the biological control activity of *Bacillus* species against a broad spectrum of fungal pathogens, such as *Aspergillus* (Cho *et al*., 2009), *Fusarium* (Lee *et al*., 2017), *Verticillium* (Han *et al*., 2015), *Alternaria* (Arrebola *et al*., 2010), *Colletotrichum* (Kim *et al*., 2010), *Phytophthora* (Park *et al*., 2016) and *Phoma* (Kalai‐Grami *et al*., 2016). Moreover, due to its low toxicity, low allergenicity, biodegradability, high stability and environmental friendliness (Meena and Kanwar, 2015), iturin A is an ideal potential biological control agent to reduce the use of chemical pesticides in agriculture. However, its application is limited mainly due to the producer strains’ low productivity and the high production costs (Meena and Kanwar, 2015). The cost of the raw material accounts for a great proportion of the total production costs in most biotechnological processes (Jain *et al*., 2013), and using low‐cost raw materials such as RSM from abundant sources is consequently an important aspect of improving the economic viability of industrial iturin A production. In a word, direct utilization of the nutrients in RSM to produce iturin A by a more efficient *Bacillus* strain in submerged fermentation seems to be an effective and economical way to realize high‐value usage of RSM and large‐scale production of iturin A.

Although protein is the main component of RSM, the meal contains carbohydrates, minerals and other nutrients (Wang *et al*., 2010; Lomascolo *et al*., 2012). However, the exact roles of protein and other components in the fermentation process have not been systematically investigated. In order to carry out basic research on the application of main nutrients in RSM to iturin A fermentation, glucose, as the most widely distributed and important reductive monosaccharide in nature, was chosen as the sole carbon source. This study evaluated the potential of integrating iturin A production with RSM utilization. The ability of a potentially industrial strain *B. amyloliquefaciens* CX‐20 to produce iturin A using RSM was identified first, followed by the optimization and simplification of the fermentation conditions. In addition, an evaluation model for the utilization of nutrients from RSM was established by analysing separate combinations of isolated protein from RSM (PRSM), fibre and inorganic salts. Based on this model, we investigated the effect of each component on iturin A production.

## Results and discussion

### Efficient utilization of the nitrogen from RSM to produce iturin A by *B. amyloliquefaciens* CX‐20

**Figure 1:**
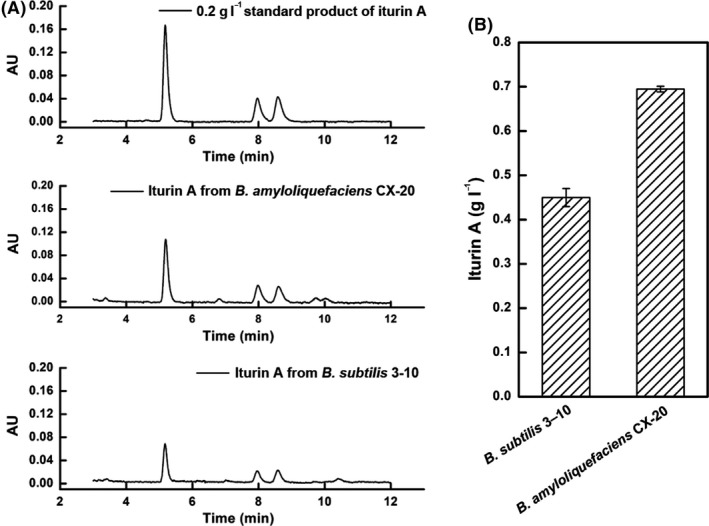
The ability of *B. amyloliquefaciens* CX‐20 to produce iturin A in RSM fermentation broth. A. HPLC spectrum of 0.2 g l^−1^ iturin A standard solution, fermentation broth of *B. amyloliquefaciens* CX‐20 and *B. subtilis* 3–10. B. Total concentrations of iturin A produced by *B. amyloliquefaciens* CX‐20 and *B. subtilis* 3–10 under the same fermentation conditions with 90 g l^−1^ initial RSM and 20 g l^−1^ initial glucose.

The feasibility of using untreated RSM as a nitrogen source for iturin A production by *B. subtilis* 3‐10 in submerged fermentation was previously evaluated by our team (Jin *et al*., 2014). However, the maximum iturin A concentration only reached 0.45 g l^−1^ after 72 h in shake flasks. In order to improve the production of iturin A, screening another more efficient strain seems to be an effective way. The iturin A production ability of *B. amyloliquefaciens* CX‐20 with RSM as nitrogen source was firstly evaluated in this study. The reported optimal initial RSM concentration for iturin A production by *B. subtilis* 3–10 was 90 g l^−1^ when used as a nitrogen source in submerged fermentation, while the optimal initial glucose concentration was 20 g l^−1^ (Jin *et al*., 2014). Therefore, to compare iturin A production by *B. amyloliquefaciens* CX‐20, an initial RSM concentration of 90 g l^−1^ and initial glucose concentration of 20 g l^−1^ were adopted at the beginning of shake flasks experiments. The results demonstrated that untreated RSM could be used by *B. amyloliquefaciens* CX‐20 to produce iturin A (Fig. 1A), and its concentration was 0.69 g l^−1^, 54.38% higher than that of *B. subtilis* 3–10 (Fig. 1B).

### Influence of initial glucose and RSM concentrations on iturin A production in shake flasks

**Figure 2:**
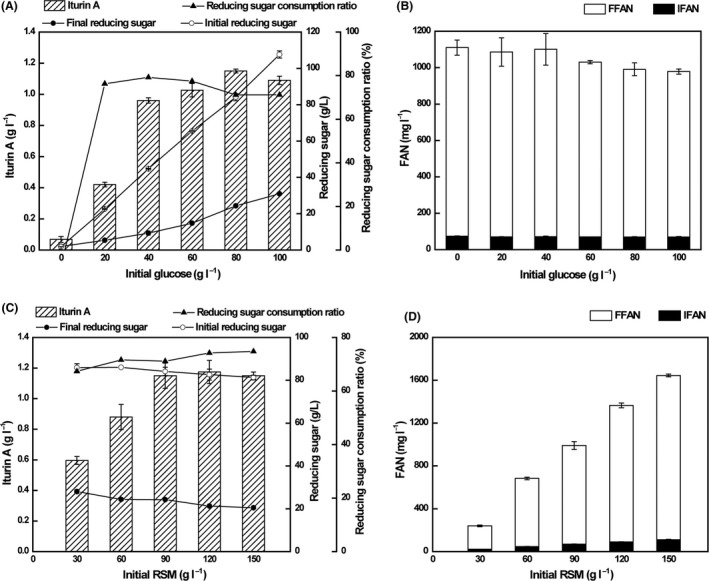
Effects of different initial glucose concentrations and RSM concentrations on iturin A production, concentrations of reducing sugars and FAN at 72 h in shake flasks. A. Effects of different initial glucose concentrations on iturin A production, concentrations of initial and final reducing sugars, and the reducing sugar consumption ratio. B. Effects of different initial glucose concentrations on the concentrations of IFAN and FFAN. C. Effects of different initial RSM concentrations on iturin A production, initial and final concentrations of reducing sugars, and the reducing sugar consumption ratio. D. Effects of different initial RSM concentrations on the concentrations of IFAN and FFAN.

Based on our previous results (Jin *et al*., 2014, 2015), the initial RSM concentration of 90 g l^−1^ was chosen to determine the effect of different initial glucose concentrations ranging from 0 to 100 g l^−1^ on iturin A production under shake flasks conditions. The results clearly demonstrated that the optimal initial glucose concentration for iturin A production was 80 g l^−1^, and the corresponding iturin A concentration reached 1.15 g l^−1^ (Fig. 2A). As a complex substrate, RSM could not only be used as a nitrogen source (Kiran *et al*., 2012; García *et al*., 2013; Chatzifragkou *et al*., 2014; Jin *et al*., 2015), but also provide a carbon source for the growth and metabolism of microorganisms (Chen *et al*., 2011; Almeida *et al*., 2015). However, when RSM was used as the carbon source, its carbon content appeared to be insufficient for supporting the fermentation and synthesis of iturin A by *B. amyloliquefaciens* CX‐20. Only 0.07 g l^−1^ iturin A was produced without initial glucose addition. With the increase in the initial glucose concentration, the production of iturin A increased first (from 0 to 80 g l^−1^) and then decreased (from 80 to 100 g l^−1^). After fermentation for 72 h, all final free ammonium nitrogen (FFAN) concentrations in the fermentation broth with different initial glucose concentrations reached about 1000 mg l^−1^, around 15 times higher than the initial free ammonium nitrogen (IFAN) concentrations (Fig. 2B). And the FFAN concentrations were not obviously changed irrespective of different initial glucose concentrations. Even without glucose supplementation, a large amount of FFAN was released from the RSM, suggesting that *B. amyloliquefaciens* CX‐20 has a good ability to hydrolyse RSM. However, the final reducing sugar concentration increased with the increased supplementation of glucose. An interesting phenomenon was that the glucose consumption efficiency reached a stable level, about 75%, at initial glucose concentrations was from 20 to 100 g l^−1^ (Fig. 2A). We speculated that this might be related to the growth of *B. amyloliquefaciens* CX‐20, although *B. amyloliquefaciens* CX‐20 growth was not positively correlated with the synthesis of iturin A. And the growth status of *B. amyloliquefaciens* CX‐20 correlates with the hydrolysis of RSM and the concentration of FFAN.

An initial glucose concentration of 80 g l^−1^ was used in the following experiments. Subsequently, the effects of different RSM concentrations ranging from 30 to 150 g l^−1^ on iturin A production were explored. As shown in Fig. 2C, with the increase in RSM concentration from 30 to 90 g l^−1^, the production of iturin A increased. However, the production reached 1.15 g l^−1^ and did not increase further after the RSM concentration was increased from 90 to 150 g l^−1^. To save costs and to achieve effective use of RSM, we chose 90 g l^−1^ as the optimal initial RSM concentration, which further validated the optimization of the 80 g l^−1^ initial glucose concentration. Although the iturin A production did not show obvious changes with the increase in RSM concentration from 90 to 150 g l^−1^, the content of the FFAN in fermentation broth after 72 h showed a positive correlation with the initial concentration of RSM (Fig. 2D), which further proved that *B. amyloliquefaciens* CX‐20 could efficiently hydrolyse RSM.

### Establishment of an evaluation model to analyse the positive roles of proteins and mineral elements in RSM for iturin A production

**Figure 3:**
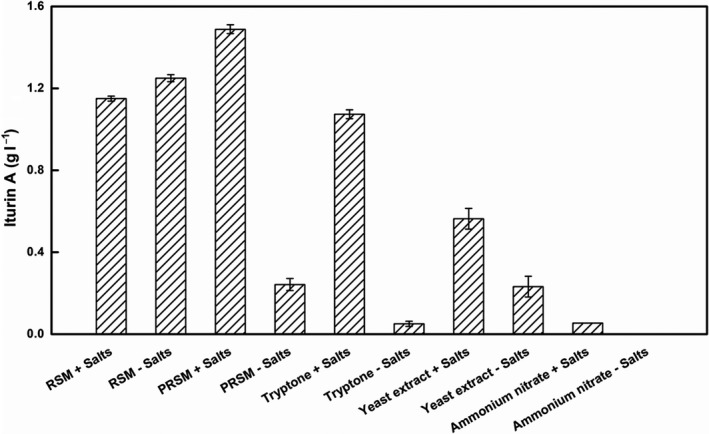
Effects of inorganic salts on iturin A production with different nitrogen sources. With 80 g l^−1^ initial glucose, the initial concentrations of RSM, PRSM, tryptone, yeast extract and ammonium nitrate were 90, 40, 22, 30 and 8 g l^−1^ respectively. Media with (+) or without (−) the addition of inorganic salts were used for iturin A production and to analyse the effects of inorganic salts on iturin A production.

RSM is rich in various mineral elements, such as calcium, phosphorus and iron (Wang *et al*., 2010; Lomascolo *et al*., 2012). However, whether the mineral elements contained in RSM could sufficiently meet the requirements of iturin A production was not investigated in detail. To evaluate the effect of minerals on the production of iturin A, the fermentation medium was simplified by omitting the addition of inorganic salts. Thus, the simplified medium only contained 80 g l^−1^ initial glucose and 90 g l^−1^ initial RSM. After 72 h of fermentation, the iturin A concentration in this medium reached 1.25 g l^−1^ or 13.89 g iturin A per kg RSM (Fig. 3), which is very close to the maximum production level reported to date, of 14 g iturin A per kg dry substrate in the medium of soya bean curd residue using *B. subtilis* RB14‐CS (Mizumoto *et al*., 2006). Compared with the 1.15 g l^−1^ iturin A obtained in the medium with added inorganic salts, the production of iturin A increased 8.70%, which indicated that the addition of inorganic salts might have a negative effect on iturin A production when RSM was used as nitrogen source.

Alkaline extraction is the most commonly used method to extract proteins from oilseed meals (Rodrigues *et al*., 2017). Isolated protein from RSM (PRSM) could not only increase the percentage of protein, but also reduce mineral elements from RSM. Based on the nutritional composition of RSM, PRSM was used as a nitrogen source to establish an evaluation model for the utilization of nutrients from RSM. The PRSM contained 89.80% of protein, while the RSM contained 39.48%. Thus, the amount of protein in 40 g l^−1^ PRSM was comparable to that in 90 g l^−1^ RSM. Consequently, 40 g l^−1^ PRSM was chosen as the nitrogen source to simulate basic nutritional composition of RSM. When inorganic salts were added to PRSM, the production of iturin A was 1.49 g l^−1^, which was higher than that produced with 90 g l^−1^ RSM. However, when inorganic salts were not added, the concentration of iturin A was greatly reduced, reaching only 0.24 g l^−1^, which was 83.73% lower than with the addition of inorganic salts (Fig. 3). As shown in Table 1, all detected mineral elements in PRSM (40 g l^−1^) were greatly reduced compared with their proportions in RSM (90 g l^−1^) at equal protein amounts. Therefore, the minerals in RSM have a great effect on iturin A production by *B. amyloliquefaciens* CX‐20.

**Table 1:** The composition of RSM hydrolysates produced with different media or microorganisms.

	Ca (mg l^−1^)	Cu (mg l^−1^)	Fe (mg l^−1^)	K (mg l^−1^)	Mg (mg l^−1^)	Mn (mg l^−1^)	P (mg l^−1^)	Si (mg l^−1^)	Zn (mg l^−1^)	FFAN (mg l^−1^)
RSM + inorganic salts	87.48	0.20	3.31	528.93	68.48	0.69	1080.92	311.65	1.04	990.94
RSM − inorganic salts	91.27	0.27	7.04	472.25	74.84	0.63	1006.53	345.60	1.35	984.56
PRSM + inorganic salts	12.54	0.01	0.32	397.32	40.10	1.95	638.79	4.44	0.12	699.79
PRSM − inorganic salts	10.69	0.01	0.16	81.34	3.71	0.08	290.32	4.13	0.11	551.85
*Aspergillus oryzae* 92011	37.65	0.59	4.69	117.45	80.31	0.64	383.17	163.11	1.33	448.46
*Trametes* sp. 48424	62.95	0.59	6.9	117.51	139.02	1.66	633.78	183.36	3.49	889.41
*Aspergillus niger* 93298	31.25	0.79	13.26	126.47	90.62	2.26	588.34	187.79	5.90	754.99

In all hydrolyses, the initial concentration of RSM or PRSM was 90 g l^−1^ or 40 g l^−1^. The protein content in 90 g RSM was equal to that of 40 g PRSM. Media with (+) or without (−) the addition of inorganic salts were used.

In order to test the effect of inorganic salts in microbial fermentation, tryptone, yeast extract and ammonium nitrate were applied in batch fermentation to compare the production performance of iturin A produced with that obtained using RSM in shake flasks. The four nitrogen sources (RSM, tryptone, yeast extract and ammonium nitrate) are typical representatives of plant, animal, microbial and inorganic nitrogen respectively. The optimal concentrations of RSM, tryptone, yeast extract and ammonium nitrate for iturin A production by *B. subtilis* 3–10 were 90, 22, 30 and 8 g l^−1^, respectively, based on our previous study (Jin *et al*., 2014). As shown in Fig. 3, RSM and tryptone were more suitable sources of nitrogen for iturin A production, which, respectively, reached 1.15 g l^−1^ and 1.07 g l^−1^ with the addition of inorganic salts. However, only small amounts of iturin A were produced when ammonium nitrate was used as the nitrogen source, which was consistent with the previous study (Jin *et al*., 2014). The iturin A concentration only reached 0.56 g l^−1^ with yeast extract as the nitrogen source, less than half of that obtained using RSM as the nitrogen source. Taken together, these results indicate that RSM has advantages as the nitrogen source for the production of secondary metabolites by *Bacillus* species.

A reduction in iturin A production was also observed when tryptone, yeast extract and ammonium nitrate with no inorganic salt supplementation were used as the nitrogen sources. Especially for tryptone without the addition of inorganic salts, the final iturin A concentration was only 0.05 g l^−1^, which was reduced more than 95% compared with that obtained with added inorganic salts. Ammonium nitrate was an inefficient nitrogen source for iturin A production, and no iturin A was detected without the addition of inorganic salts. In the case of yeast extract, although removing inorganic salts from the culture medium also reduced the production of iturin A, it only decreased from 0.56 g l^−1^ to 0.23 g l^−1^. As can be seen in Fig. 3, inorganic salts were necessary for *B. amyloliquefaciens* CX‐20 to produce iturin A, and the mineral elements in RSM, which could be used as a substitute for inorganic salts, had a positive effect on iturin A production. All these results indicated that untreated RSM could be used as the sole source of all nutrients except the carbon source for the production of iturin A using *B. amyloliquefaciens* CX‐20.

### Usage of the established evaluation model to predict the nitrogen potential of RSM for iturin A production

**Figure 4:**
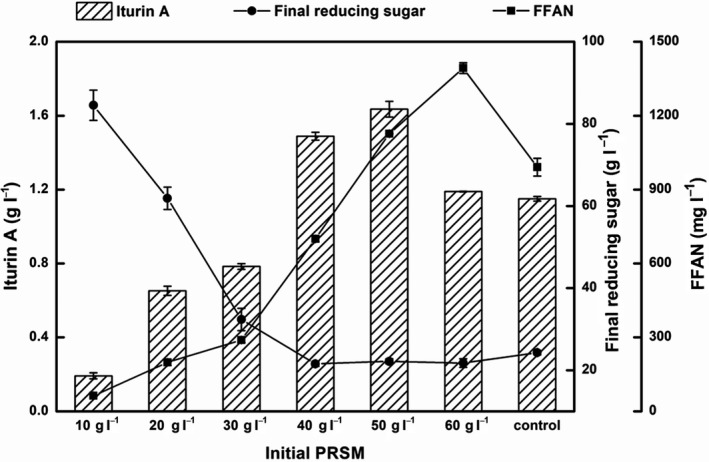
Effects of different initial PRSM concentrations on iturin A production, concentrations of final reducing sugars and FFAN. With 80 g l^−1^ initial glucose, the initial concentrations of PRSM were ranged from 10 to 60 g l^−1^. Medium containing 80 g l^−1^ initial glucose, 90 g l^−1^ initial RSM and inorganic salts was taken as the control.

As shown in Fig. 3, although the production of iturin A in the PRSM medium (1.49 g l^−1^) was higher than in the RSM medium (1.15 g l^−1^) with the same protein amount and the addition of inorganic salts, the extraction of protein from RSM using the alkali method was not only time‐consuming, tedious and costly, but also caused the loss of mineral elements and some of the protein in RSM. Nevertheless, this model of RSM nutrients was able to partially predict the minimum yield of iturin A produced by *B. amyloliquefaciens* CX‐20 when the proteins in RSM were more fully utilized, excluding effects of other factors, especially the physical and chemical structures that hinder the assimilation of proteins from RSM by microorganisms (Ramachandran *et al*., 2007; Wang *et al*., 2010). Therefore, the optimal concentration of PRSM for iturin A production was investigated. Medium containing 80 g l^−1^ initial glucose, 90 g l^−1^ initial RSM and inorganic salts was taken as the control. As shown in Fig. 4, with the addition of inorganic salts, the iturin A production was enhanced with the increase in PRSM concentration from 10 to 50 g l^−1^, reaching the maximum concentration of 1.64 g l^−1^, which was 42.20% higher than that of the control (1.15 g l^−1^). Taking into account the protein content of RSM and PRSM (39.48 and 89.80% respectively), the percentage of protein in 50 g PRSM was equivalent to that in 113.73 g RSM, which was in the optimal initial RSM concentration range for iturin A production of 90–150 g l^−1^. However, under the conditions of equal protein content, the yield of iturin A obtained from PRSM was still higher than that obtained from RSM. This phenomenon is probably caused by a combination and interaction of other components of RSM such as fibre, non‐starch polysaccharides (de Vries *et al*., 2014) or phytate (Haese *et al*., 2017; Rodrigues *et al*., 2017). Further pretreatment, which could make the nutrients in RSM more accessible, might be an effective way to improve the RSM utilization (Chen *et al*., 2011; Chatzifragkou *et al*., 2014).

It was reported that the solubility of rapeseed protein was relatively low (Bos *et al*., 2007), and the concentration of soluble proteins and IFAN in the media with different concentrations of PRSM were therefore very low. However, after 72 h of fermentation, the concentration of FFAN increased with the increase in the initial PRSM concentration. When the initial concentration of PRSM was 50 g l^−1^ or more, its corresponding concentrations of FFAN were higher than those of the control. The final reducing sugar concentration decreased with the increase in the initial PRSM concentration until it reached 40 g l^−1^, and final reducing sugar concentration reached a stable level. These results proved that proteins in RSM have the greatest effect on the production of iturin A.

**Figure 5:**
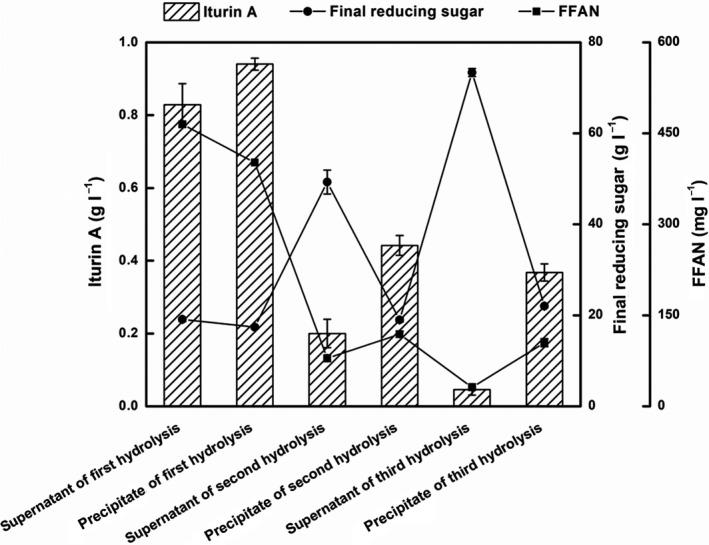
Effects of three batches of hydrolysed supernatants and precipitates from RSM on iturin A production, concentrations of final reducing sugars and FFAN. The first, second and third batch media were named as supernatant of first hydrolysis and precipitate of first hydrolysis, supernatant of second hydrolysis and precipitate of second hydrolysis, supernatant of third hydrolysis and precipitate of third hydrolysis, respectively.

We speculated that a nitrogen potential of RSM for iturin A production higher than 1.64 g l^−1^ could be achieved based on the following preconditions: (i) The protein was not completely extracted from RSM by the alkali method, and the protein potential for iturin A production had not been fully utilized. The extraction rate of protein was significantly reduced with the increase in extraction time, which was reflected in the production of iturin A in different batch supernatants, from 0.83 g l^−1^ to 0.05 g l^−1^ (Fig. 5). As expected, the amount of iturin A produced by the precipitate fermentation of all hydrolysates was higher than that obtained using the supernatants from the same batch, although the total amount of iturin A production from the supernatant and precipitate fermentation from the same batch was still less than that produced by the precipitate of the previous batch. The iturin A production by supernatant fermentation was lesser than that obtained by precipitate fermentation, and the difference increased with the extension of hydrolysis time. After three rounds of hydrolysis, the production of iturin A obtained by supernatant fermentation was almost negligible, which further confirmed that the extraction rate of RSM protein would be significantly reduced to near zero after threefold alkaline extraction. However, even after triple extraction, there was still some protein remaining in the precipitate that could be potentially used for iturin A production (0.37 g l^−1^). The content of the FFAN in either the supernatant or the precipitate decreased with the extension of hydrolysis time, while the final reducing sugar concentration had an opposite change trend. This indicated a significant correlation between carbon sources and nitrogen sources in fermentation process. (ii) A part of the nitrogen from PRSM was not fully utilized. After 72 h of fermentation, the concentration of FFAN still remained at 1126.58 mg l^−1^, in addition to some undissolved protein (Fig. 4). The optimization of fermentation process parameters, such as the carbon source feeding strategy, repeated batch fermentation or pretreatment (Kiran *et al*., 2012, 2013; García *et al*., 2013; Chatzifragkou *et al*., 2014; Jin *et al*., 2015), would further improve the utilization of nutrients in RSM and increase the yield of iturin A. Moreover, it has been confirmed in our laboratory that other carbon sources, such as molasses and glycerol, could further increase the yield of iturin A (data not shown).

### The negative roles of fibres in RSM for iturin A production

**Figure 6:**
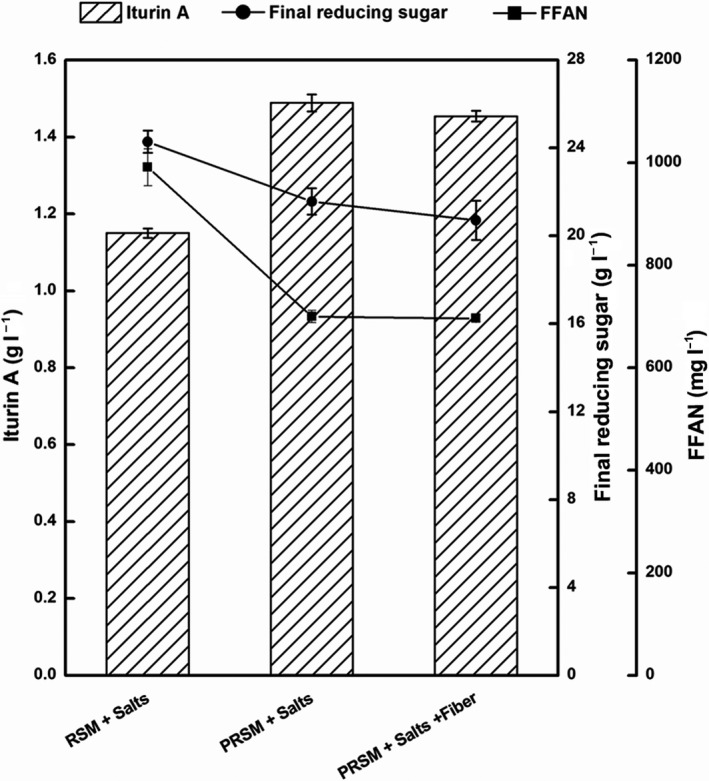
Effects of fibre on iturin A production, concentrations of final reducing sugars and FFAN. With 80 g l^−1^ initial glucose, the initial concentrations of RSM, PRSM and fibre were 90, 40 and 11 g l^−1^ respectively.

The next step was to determine the effects of the fibre in RSM on iturin A production (Fig. 6). The content of crude fibre in RSM was 12%, so 11 g l^−1^ fibre was added into the culture medium which also contained 40 g l^−1^ PRSM and inorganic salts. The results showed that the addition of fibre could slightly reduce the yield of iturin A, but the produced amount was still higher than that of the RSM medium. Therefore, we speculated that (i) the crude fibre in RSM might prevent *B. amyloliquefaciens* CX‐20 from utilizing other nutrients in RSM and (ii) the release and separation of nutrients in RSM might promote the yield of iturin A.

### The nutrients in RSM released with increased solubility by the fermentation of *B. amyloliquefaciens* CX‐20

It has been extensively reported that the nutrients of RSM could be made accessible for microorganisms when it was treated by short‐term solid‐state fungal fermentation followed by enzymatic hydrolysis (Kiran *et al*., 2012, 2013; García *et al*., 2013; Chatzifragkou *et al*., 2014). In order to evaluate the advantages of submerged fermentation by *B. amyloliquefaciens* CX‐20 to hydrolyse RSM, three fungi that were preferentially used to hydrolyse RSM by SSF in previous reports (Kiran *et al*., 2012, 2013; García *et al*., 2013; Chatzifragkou *et al*., 2014) were chosen as controls. *A. oryzae* 92011 and *A. niger* 93298 are moulds that were previously characterized as efficient producers of predominantly proteases and, to a lesser extent, of phytase, cellulase, xylanase and amylolytic enzymes (Adav *et al*., 2010; Shi *et al*., 2016). *Trametes* sp. 48424 is a white‐rot fungus with high laccase activity and therefore has the important ligninolytic enzymes responsible for lignin degradation (Fan *et al*., 2011; Niu *et al*., 2015). After 72 h of SSF, fermented RSM at the concentration of 90 g l^−1^ was hydrolysed for 24 h at 55°C, and the composition of the mixture of the hydrolysate was analysed (Table 1). As expected, whether inorganic salts were added or not, the concentrations of free ammonium nitrogen (FAN) and some soluble metal ions and other minerals, especially Ca, K, P and Si, in the fermentation broth of *B. amyloliquefaciens* CX‐20 were much higher than those in the hydrolysates produced by the three fungi after 72 h of fermentation. The content of phosphorus in RSM was 1.33%, corresponding to 1197 mg l^−1^ phosphorus from the culture medium with 90 g l^−1^ RSM. However, most of the phosphorus was insoluble (Vig and Walia, 2001). An interesting phenomenon was that the content of soluble phosphorus in the fermentation broth reached more than 1000 mg l^−1^ after fermentation by *B. amyloliquefaciens* CX‐20, which suggested that most of the phosphorus in RSM was hydrolysed from insoluble phytic acid to yield the soluble form. Phytate is the major storage form of phosphorus in most cereal grains and oilseeds, and it usually accounts for 60–90% of the total available phosphorus. Beside its ability to chelate and precipitate minerals, phytate is also closely associated with proteins, especially in oilseeds, decreasing the bioavailability of critical nutrients such as protein, calcium, magnesium, zinc and iron (Haese *et al*., 2017; Rodrigues *et al*., 2017). Therefore, we predicted that the content of soluble phosphorus in the hydrolysate could reflect the hydrolysis ability of *Bacillus* for RSM to a great extent. However, the hydrolysis ability cannot be judged only by the concentration of FFAN because it would also be affected by the consumption demands for microbial growth and metabolism.

### RSM nutrient cycling and efficient resource utilization by *B. amyloliquefaciens* CX‐20 fermentation

**Figure 7:**
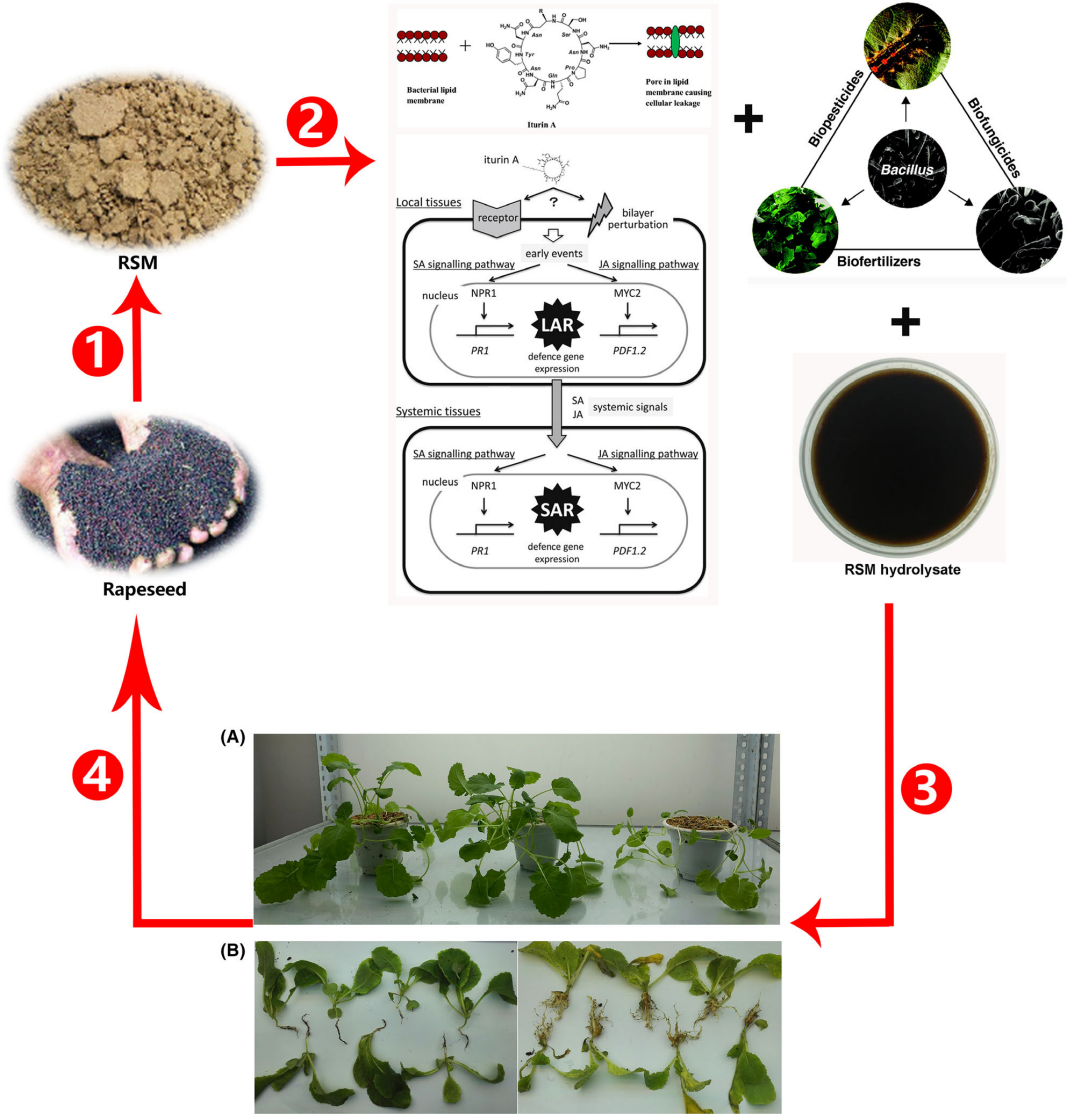
Schematic diagram of nutrient cycling and efficient resource utilization of RSM. There were four steps as follows: (1) Rapeseed was processed to obtain agricultural by‐products – RSM. (2) After the submerged fermentation by *B. amyloliquefaciens* CX‐20, RSM was converted into the combination of Bacillus, iturin A and RSM hydrolysate. (3) The mixture of Bacillus, iturin A and RSM hydrolysate simultaneously combatted clubroot disease and promoted the growth of *Brassica napus*. A. The function of the mixture as biofertilizer on promoting the growth of *Brassica napus*. The *Brassica napus* from left to right grew for 40 days after treated with the fermentation broth diluted by 500 or 50 times, and the control sprayed only with water respectively. B. The function of mixture as biological control on combatting clubroot disease. The Chinese cabbage was grown in soli containing pathogenic microorganisms of clubroot disease. The left samples were treated with 50 times diluted fermentation broth, and the right samples were the controls sprayed only with water. (4) The output of rapeseed increased.

The traditional method of composting oilseed meal as fertilizer is still practiced in many parts of China and other developing countries. However, this method has some drawbacks, such as a high loss of nitrogen, environmental pollution and a long fermentation period (1–6 months). This ineffective and insufficient utilization of nitrogen by plant would cause significant waste. SSF has been widely used to hydrolyse meal protein (Wang *et al*., 2014). However, conditions during the SSF process are difficult to be controlled, which limits the large‐scale industrialization of this procedure (Mitchell *et al*., 2003; Holker *et al*., 2004). Our study demonstrated that the methodology of submerged fermentation of RSM by *B. amyloliquefaciens* CX‐20 had several advantages such as the much shorter period (only 3 days), easily monitored and controlled process. But most notably, this method could not only produce iturin A and live *Bacillus* by directly using the nutrients in RSM, but also hydrolyse insoluble proteins and mineral elements in RSM to make them water‐soluble, as shown in Fig. 7. Biofertilizer, especially water‐soluble fertilizer containing free amino acids such as RSM hydrolysate, has been widely used to promote the growth of plants and improve their quality (Wang *et al*., 2014). *Bacillus*, marketed as microbial pesticides, fungicides or fertilizers, has offered many advantages for their application in agricultural biotechnology (Pérez‐García *et al*., 2011). Iturin A, a prominent member of the iturin group, was found to suppress many plant diseases via a combination of its broad‐spectrum antifungal activity and the activation of plant defence systems (Han *et al*., 2015; Kawagoe *et al*., 2015). The mixture of *Bacillus*, iturin A and RSM hydrolysate could be used as biological control to combat clubroot disease of *Brassica napus* (Fig. 7B). Clubroot disease, caused by *Plasmodiophora brassicae*, threatens *Brassicaceae* crop production worldwide, which resulted in major economic losses (Hirani *et al*., 2018). And there have been no reports on whether iturin A could inhibit clubroot disease. In order to simplify the process and save costs, we did not isolate and purify iturin A from fermentation broth; the fermentation broth contained 1.25 g l^−1^ iturin A was directly used after dilution. As shown in Fig. 7B, Chinese cabbage was grown in soil containing *P. brassicae* of clubroot disease. Chinese cabbage grew better in flowerpots sprayed with 50 times diluted fermentation. Compared with the control group sprayed only with water, the root was normal and showed no sign of disease. As shown in Fig. 7A, the mixture of *Bacillus*, iturin A and RSM hydrolysate could be also used as biofertilizer to promote the growth of *Brassica napus*. The fermentation broth diluted by 50 and 500 times had obvious growth‐promoting effect on *Brassica napus*. Compared with the control group sprayed only with water, the plant size had obvious advantages. In a word, RSM nutrient cycling and efficient resource utilization by *B. amyloliquefaciens* CX‐20 submerged fermentation in this study is significant to promote the rapeseed production and valorization.

In summary, RSM was first proved to be used as the sole source of all nutrients except the carbon source for the high productivity of iturin A by industrial strain *B. amyloliquefaciens* CX‐20. Moreover, a fermentation model was established to analyse the specific roles of different RSM components on iturin A production. Proteins and minerals in RSM were firstly proved to play positive role, whereas fibre had negative effect. The maximal concentration of iturin A was predicted to be more than 1.64 g l^‐1^ by the established evaluation model, which was higher than the maximum production level reported to date. In conclusion, submerged fermentation of *B. amyloliquefaciens* CX‐20 to hydrolyse RSM and release water‐soluble nutrients was demonstrated as a promising strategy to achieve full utilization of RSM to produce a combination of value‐added biological control agent and biofertilizer.

## Experimental procedures

### Microorganisms and media

The iturin A production strains *B. subtilis* 3‐10 (GenBank accession number JF460845) and *B. amyloliquefaciens* CX‐20 (CCTCC NO: M 2018794) were kindly provided by Professor Shouwen Chen (College of Life Sciences, Hubei University, Wuhan, China). Luria‐Bertani (LB) medium comprising (per litre) 10 g tryptone, 5 g yeast extract and 10 g NaCl was used for seed cultures of *Bacillus*. The fermentation medium was composed of (per litre) 80 g Glucose, 1 g K_2_HPO_4_·3H_2_O, 0.5 g MgSO_4_·7H_2_O, 0.005 g MnSO_4_·H_2_O and 90 g RSM, which was provided by Oil Crops Research Institute of Chinese Academy of Agricultural Sciences (Wuhan, China). The medium was adjusted to pH 7.0 and autoclaved at 121°C for 30 min. Shake‐flask fermentations were performed in 250‐ml flasks containing 20 ml of medium at 28°C and 220 r.p.m. The inoculation size was 5% (v/v).

*Trametes* sp. 48424 (Zhang *et al*., 2016) was obtained from the School of Life Science and Technology, Huazhong University of Science and Technology. *A. oryzae* 92011 (CCTCC NO: AF 92011) and *A. niger* 93298 (CCTCC NO: AF 93298) were obtained from the China Center for Type Culture Collection. These three fungi were employed in SSF to produce crude enzymes essential for RSM hydrolysis. The strains were maintained in the form of spores, kept in dry sand at 4°C. For the preparation of inocula for SSF, spores were purified and sporulated in slat tubes of medium containing 25 g l^−1^ RSM, 25 g l wheat bran and 20 g l^−1^ agar.

### Extraction of proteins from RSM using alkali

The RSM was ground to pass through a 120‐mesh screen and then dispersed in deionized water (1:12 w/v), adjusted to pH 12.0 with 1 M sodium hydroxide and stirred at 55°C for 40 min. The slurry was centrifuged at 4000 r.p.m. for 10 min, the supernatant recovered, and the meal re‐extracted twice (1:10 w/v and 1:8 w/v respectively) as described above. The resulting supernatants were combined, and the resulting solution was adjusted to pH 4.5 with 1 M citric acid and centrifuged at 4000 r.p.m. for 10 min again, after which the precipitate was rinsed with water once and lyophilized for further use.

### Effects of three batches of hydrolysed supernatants and precipitates from RSM on iturin A production

An initial 90 g l^−1^ RSM was dissolved in water, after which the pH was adjusted to 12 with 1 M sodium hydroxide and the mixture was stirred at 55°C for 2 h. The slurry was centrifuged at 4000 r.p.m. for 10 min. The supernatant was separated, and an equal volume of water was added to the precipitate. Then, the pH of the supernatant and precipitate slurry was adjusted to 7 with 1 M hydrochloric acid. After supplementation with 90 g l^−1^ glucose and inorganic salts, these two media were named as the supernatant of first hydrolysis and precipitate of first hydrolysis respectively. The precipitate, acquired from the first round of hydrolysis, was further re‐hydrolysed using the same operation as above, and the second batch media were named as supernatant of second hydrolysis and precipitate of second hydrolysis. Similarly, the third batch media were named as supernatant of third hydrolysis and precipitate of third hydrolysis.

### SSF followed by fungi autolysis

SSF was employed by using three fungi *Trametes* sp. 48424, *A. oryzae* 92011 and *A. niger* 93298 for the subsequent hydrolysis of RSM respectively. Prior to each SSF, three fungi *Trametes* sp. 48424, *A. oryzae* 92011 and *A. niger* 93298 were sporulated on the surface of a solid medium, containing 25 g l^−1^ RSM, 25 g l^−1^ wheat bran and 20 g l^−1^ agar, in 20‐ml test tubes incubated for 5 days at 28°C. Then, three aqueous spore suspensions were formed by adding 10 ml of sterile distilled water with 0.01% Tween 80 (v/v) in each test tube. These spore suspensions served as inocula for SSF, which were performed in pre‐sterilized (121°C for 30 min) 250‐ml flasks containing 1.8 g of RSM as the sole source of nutrients. The moisture contents were adjusted to 65% (w/w) after inoculation of fungi spores. All flasks were incubated with approximately 10^6^ spores/g RSM at 28°C for 72 h.

Autolysis of fermented solids was subsequently conducted by mixing the distilled water to final volume of 20 ml so that initial RSM concentrations were approximately 90 g l^−1^. The content was blended and incubated at 55°C for 24 h in flasks.

### Effects of the RSM fermentation broth by *B. amyloliquefaciens* CX‐20 on the growth of *Brassica napus* and combatting clubroot disease

*Brassica napus* and Chinese cabbage seeds were kindly provided by Oil Crops Research Institute of the Chinese Academy of Agricultural Sciences, Wuhan, China. The seeds were surface‐sterilized in 1% sodium hypochlorite for 5 min, washed with distilled water and germinated on moistened filter paper for 7 days. The resulting seedlings were used in all inoculation experiments. *Brassica napus* roots with galls developing from natural infections by *P. brassicae* were collected from an experimental field plot near Wuhan, China. The galls were kept at −20°C, and resting spores from these galls were used as the initial inoculum (Feng *et al*., 2013).

Sea sand was washed with distilled water, autoclaved at 121°C for 60 min and then dried at 80°C overnight. For each inoculation, 4.5 volumes of sand were mixed with 1 volume of water or spore suspension to saturate the sand. Based on the post‐inoculation volume, the concentrations of spore suspensions were adjusted to 10^7^ resting spores per ml. Throughout this study, all plant inoculations were conducted by transplanting pre‐germinated seedlings into the inoculated sand (Feng *et al*., 2013). Then, 50 ml extra diluted fermentation broth or water was added into each flowerpot. The growth cycles of Chinese cabbage and *Brassica napus* were set at 20 and 40 days respectively.

### Analytical methods

Iturin A was extracted according to a reported method (Jin *et al*., 2014). Briefly, 0.3 ml of the mixed fermentation broth was added into a 2‐ml glass tube containing 1.2 ml of methanol, shaken well and incubated at room temperature for 60 min. The mixture was centrifuged at 12 000 r.p.m. for 20 min, and the supernatant was filtered through a 0.22 μm pore‐size hydrophobic polytetrafluoroethylene (PTFE) type disposable syringe. The iturin A concentration was quantified using a Waters 2695 HPLC system equipped with an ACQUITY UPLC BEA C18 column (1.7 μm 2.1 × 100 mm, Waters, Milford, MA, USA). A mixture of acetonitrile and 10 mM ammonium acetate (35:65, v/v) was used as the mobile phase at a flow rate of 0.3 ml min^−1^, and the elution was monitored at 210 nm. The concentration of iturin A was analysed and quantified using an authentic reference standard (Sigma Chemicals, St. Louis, MO, USA, CAS: 52229‐90‐0). The content of iturin A was measured using triplicate samples.

The concentrations of reducing sugar in the fermentation were determined using the DNS method using 3, 5‐dinitrosalicylic acid reagent (Miller, 1959). The concentration of free ammonium nitrogen (FAN) was determined using the ninhydrin colorimetric method (Lie, 1973). The concentrations of Ca, Cu, Fe, K, Mg, Mn, P, Si and Zn were measured using Agilent's 5110 Synchronous Vertical Dual View (SVDV) ICP‐OES system (Agilent Technologies, Santa Clara, CA, USA).

All experiments were performed in triplicate, and the data were processed using Origin v8.6 software (Origin Lab Corp., Northampton, MA, USA).

## Conflicts of interest

None declared.
